# Hippocampal Neurons Keep Track of Two Things—One Moving, One Not—Simultaneously

**DOI:** 10.1371/journal.pbio.1000404

**Published:** 2010-06-22

**Authors:** Richard Robinson

**Affiliations:** Freelance Science Writer, Sherborn, Massachusetts, United States of America

**Figure pbio-1000404-g001:**
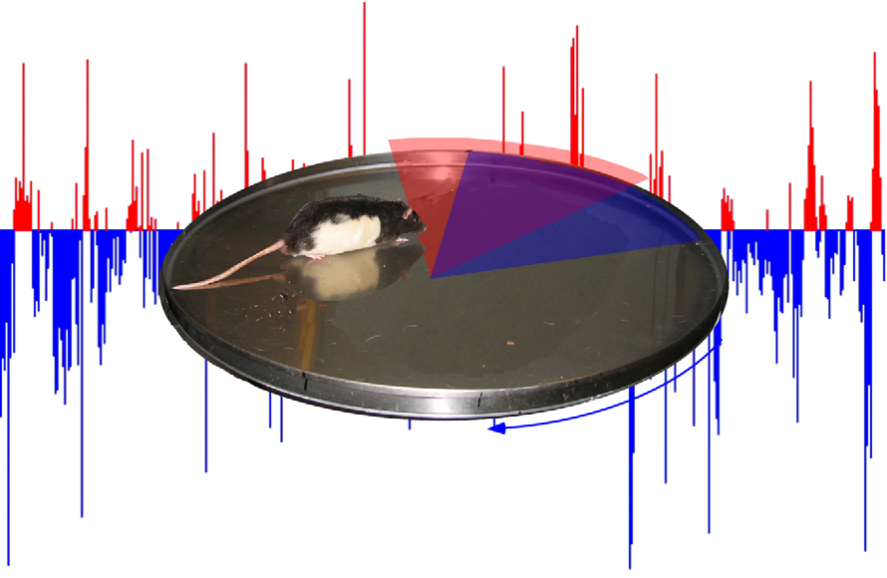
Cognitive control of distinct streams of information is at the core of understanding how we think, but how the electrical activity of neurons mediates cognitive control is unknown.

[Fig pbio-1000404-g001]Animals move, and because they do, they must be masters of multiple frames of reference. As the monkey vaults himself through the air, his eye must be on the stationary branch looming into view; as the child circles on the merry-go-round, her eye must track the brass ring just around the curve.

Each frame provides its own stream of spatial information—the moving limb, the fixed target—and processing both streams, while keeping the two separate, is the key to successful behavior in a complex world. This ability, called cognitive control, is clearly essential, but the neural mechanisms underlying it are not well understood. One tool used in cognitive control is believed to be “dynamic grouping,” in which a single set of neurons fires in different patterns, each representing a different information stream, and each pattern alternates in quick succession to allow the brain to process each stream without mashing up the information each contains.

While some studies have suggested the hippocampus—a center not only of memory creation but also of spatial positioning—engages in dynamic grouping, the evidence has been conflicting. In a study in this issue, Eduard Kelemen and André Fenton show that the hippocampus does indeed use dynamic grouping to process dual (and dueling) spatial information streams.

To present a rat with two different spatial frames of reference simultaneously, the authors placed the rat within a rotating “arena,” in which it could scurry around to pick up food rewards. The arena's floor was marked with two wedge-shaped zones, each of which would deliver a shock if the rat stepped on it. One wedge was stationary with respect to the room, and so as the arena rotated and the rat was carried around the circle, it would be swept into the shock zone unless it moved away. Here, the stationary frame of reference of the room (which the rat could see beyond the arena's walls) was most relevant for avoiding the shock. The other wedge moved with the arena, and for avoiding that shock as it navigated the arena, smells and marks on the arena from a moving frame of reference were most useful.

The rat quickly learned to avoid both shocks as it negotiated this diabolical merry-go-round—the average rat improved its performance 15-fold within 5 training sessions, confirming the ability to use the two spatial frames of reference concurrently. The hippocampus was crucial for this skill; when the authors temporarily blocked one side of the hippocampus with a toxin, the rat could no longer avoid the shock zones when one was in motion.

Within the rat hippocampus, “place cells” process the spatial information that allows the rat to know where it is and to navigate its environment. To investigate the role of hippocampal neuronal ensembles in the rat's behavior, the authors recorded the firings of multiple neurons in the hippocampus while the rat navigated the rotating arena. Based on their previous work correlating discharge patterns with the positional information contained within them, the authors classified individual neuronal discharges at any moment as being stationary frame-specific (meaning the neuron was involved in processing spatial information regarding the stationary frame), or rotational frame-specific.

They found that most of the cells they recorded fired in response to one frame or another, about equally divided between the two frames. Cells that fired close together tended to be firing in response to the same frame, linking them into a frame-specific ensemble. Any individual cell could be part of either ensemble, and many were in fact part of both—they fired when either ensemble was active. In keeping with the need to process both streams of information simultaneously, the room-specific ensemble and the arena-specific ensemble alternated their firing. Preference for each ensemble was greatest when the rat was closest to the relevant shock wedge—that is, the room-specific ensemble activated most when the rat was closest to the stationary wedge, versus the arena-specific ensemble when closest to the moving wedge.

Taken together, these results indicate that neuronal ensembles encoding spatial information alternate in their activity in synchrony with the relevance of the information, thus using dynamic grouping in the process of cognitive control. These results are valuable not only for understanding the hippocampus, but likely other brain regions as well, since the same challenges, and likely similar solutions, occur elsewhere in the brain. They may also provide some insight into schizophrenia, a central aspect of which is difficulty with processing multiple streams of information simultaneously.

**Kelemen E, Fenton AA (2010) Dynamic Grouping of Hippocampal Neural Activity during Cognitive Control of Two Spatial Frames. doi:10.1371/journal.pbio.1000403**

